# Cardiovascular disease, obesity, and type 2 diabetes in children born after assisted reproductive technology: A population-based cohort study

**DOI:** 10.1371/journal.pmed.1003723

**Published:** 2021-09-07

**Authors:** Emma Norrman, Max Petzold, Mika Gissler, Anne Lærke Spangmose, Signe Opdahl, Anna-Karina Henningsen, Anja Pinborg, Aila Tiitinen, Annika Rosengren, Liv Bente Romundstad, Ulla-Britt Wennerholm, Christina Bergh

**Affiliations:** 1 Department of Obstetrics and Gynecology, Institute of Clinical Sciences, Sahlgrenska Academy, University of Gothenburg, Sahlgrenska University Hospital/Östra, Gothenburg, Sweden; 2 School of Public Health and Community Medicine, Institute of Medicine, University of Gothenburg, Gothenburg, Sweden; 3 Information Services Department, Finnish Institute for Health and Welfare, Helsinki, Finland; 4 Department of Neurobiology, Care Sciences and Society, Karolinska Institute, Stockholm, Sweden; 5 Fertility Clinic, Copenhagen University Hospital, Rigshospitalet, Copenhagen, Denmark; 6 Department of Public Health and Nursing, Norwegian University of Science and Technology, Trondheim, Norway; 7 Department of Obstetrics and Gynecology, University of Helsinki, Helsinki University Hospital, Helsinki, Finland; 8 Department of Molecular and Clinical Medicine, University of Gothenburg, Sahlgrenska University Hospital/Östra, Gothenburg, Sweden; 9 Spiren Fertility Clinic, Trondheim, Norway; 10 Reproductive Medicine, Department of Obstetrics and Gynaecology, Institute of Clinical Sciences, Sahlgrenska Academy, University of Gothenburg, Sahlgrenska University Hospital, Gothenburg, Sweden; Chinese University of Hong Kong, CHINA

## Abstract

**Background:**

Some earlier studies have found indications of significant changes in cardiometabolic risk factors in children born after assisted reproductive technology (ART). Most of these studies are based on small cohorts with high risk of selection bias. In this study, we compared the risk of cardiovascular disease, obesity, and type 2 diabetes between singleton children born after ART and singleton children born after spontaneous conception (SC).

**Methods and findings:**

This was a large population-based cohort study of individuals born in Norway, Sweden, Finland, and Denmark between 1984 and 2015. Data were obtained from national ART and medical birth registers and cross-linked with data from national patient registers and other population-based registers in the respective countries. In total, 122,429 children born after ART and 7,574,685 children born after SC were included. Mean (SD) maternal age was 33.9 (4.3) years for ART and 29.7 (5.2) for SC, 67.7% versus 41.8% were primiparous, and 45.2% versus 32.1% had more than 12 years of education. Preterm birth (<37 weeks 0 days) occurred in 7.9% of children born after ART and 4.8% in children born after SC, and 5.7% versus 3.3% had a low birth weight (<2,500 g). Mean (SD) follow-up time was 8.6 (6.2) years for children born after ART and 14.0 (8.6) years for children born after SC. In total, 135 (0.11%), 645 (0.65%), and 18 (0.01%) children born after ART were diagnosed with cardiovascular disease (ischemic heart disease, cardiomyopathy, heart failure, or cerebrovascular disease), obesity or type 2 diabetes, respectively. The corresponding values were 10,702 (0.14%), 30,308 (0.74%), and 2,919 (0.04%) for children born after SC. In the unadjusted analysis, children born after ART had a significantly higher risk of any cardiovascular disease (hazard ratio [HR] 1.24; 95% CI 1.04–1.48; *p =* 0.02), obesity (HR 1.13; 95% CI 1.05–1.23; *p =* 0.002), and type 2 diabetes (HR 1.71; 95% CI 1.08–2.73; *p =* 0.02). After adjustment, there was no significant difference between children born after ART and children born after SC for any cardiovascular disease (adjusted HR [aHR]1.02; 95% CI 0.86–1.22; *p =* 0.80) or type 2 diabetes (aHR 1.31; 95% CI 0.82–2.09; *p =* 0.25). For any cardiovascular disease, the 95% CI was reasonably narrow, excluding effects of a substantial magnitude, while the 95% CI for type 2 diabetes was wide, not excluding clinically meaningful effects. For obesity, there was a small but significant increased risk among children born after ART (aHR 1.14; 95% CI 1.06–1.23; *p =* 0.001). Important limitations of the study were the relatively short follow-up time, the limited number of events for some outcomes, and that the outcome obesity is often not considered as a disease and therefore not caught by registers, likely leading to an underestimation of obesity in both children born after ART and children born after SC.

**Conclusions:**

In this study, we observed no difference in the risk of cardiovascular disease or type 2 diabetes between children born after ART and children born after SC. For obesity, there was a small but significant increased risk for children born after ART.

**Trial registration number:**

ISRCTN11780826.

## Abstract 2

Emma Norrman and co-workers study the health of children born with and without assisted reproductive technology.

## Author summary

**Why was this study done?:**

- Earlier studies have found indications of alterations in cardiometabolic risk factors in children born after assisted reproductive technology (ART).
- Most earlier studies are based on small cohorts with high risk of selection bias. Furthermore, most earlier studies have measured markers and risk factors for disease instead of clinical diseases.
- In this study we wanted to compare the risk of cardiovascular disease, obesity, and type 2 diabetes between singleton children born after ART and singleton children born after spontaneous conception (SC).

**What did the researchers do and find?:**

- By combining high-quality Nordic registers, we included singleton children born after ART (*n =* 122,429) and SC (*n =* 7,574,685) between 1984 and 2015 in Norway, Sweden, Finland, and Denmark.
- The findings showed that children born after ART had no increased risk of cardiovascular disease or type 2 diabetes after adjustment for measured confounders. For any cardiovascular disease, the 95% CI was reasonably narrow, excluding effects of a substantial magnitude, while the 95% CI for type 2 diabetes was wide, not excluding clinically meaningful effects.
- We observed a slightly increased risk of obesity in children born after ART.

**What do these findings mean?:**

- Since type 2 diabetes and most of the cardiovascular diseases had a limited number of events, the ART population is relatively young, and there was a slightly increased risk of obesity in children born after ART, there is a need for further studies with longer follow-up in this area.

## Introduction

The use of assisted reproductive technology (ART) has increased significantly during the last decades, with more than 390,000 children born worldwide annually [1] and the total number of children born after ART now exceeding 9 million [2]. Long-term cardiovascular and metabolic risks in children born after ART have gained increased attention in both animal and human studies during the last decade. Impaired activity of enzymes related to fatty acid metabolism [3], altered glucose parameters [4,5], and elevated systolic blood pressure [6,7] have been found in studies of mice born after ART. Similarly, studies in humans have found altered glucose metabolism [8,9], elevated blood pressure [8–12], increased thickness of carotid intima–media [13,14], and suboptimal cardiac diastolic function [15,16] in children born after ART. A few studies have also found significantly more peripheral body fat deposits in children born after ART [17,18]. These results have been summarized in systematic reviews and meta-analyses [19,20].

A major problem is that most published studies are based on small cohorts (14 to 2,603 ART individuals [8–16,21]) and also low participation rates (between 31% and 64% loss of contacted individuals [8,9,22]), leading to high risk of selection bias. By combining high-quality Nordic registers, we established a large cohort of children born after ART that made it possible to compare the risk of cardiovascular disease, obesity, and type 2 diabetes in singleton children and young adults born after ART, including specific ART treatments, to risk in singletons born after spontaneous conception (SC).

## Methods

### Study design and data collection

This was a population-based cohort study carried out in Norway, Sweden, Finland, and Denmark. Data were obtained from the CoNARTaS cohort (Committee of Nordic Assisted Reproductive Technology and Safety) and then cross-linked with data from national patient registers (NPRs), national cause of death registers (CDRs), SWEDIABKIDS (Swedish childhood diabetes register), the Swedish National Diabetes Register (NDR), the Swedish Prescribed Drug Register (SPDR), and BORIS (Swedish childhood obesity treatment register). Socioeconomic data were retrieved from the statistic bureaus in each country. Major birth defects, registered during the first year of birth, were defined according to the EUROCAT classification system [23]. Congenital heart defects were defined according the International Statistical Classification of Diseases and Related Health Problems (ICD) if the child had a diagnosis according to ICD-10 codes Q20–Q26 or ICD-9 codes 745–747 (excluding minor defects according to EUROCAT). Paternal data were not available. The unique personal identity number (PIN), assigned to each resident in the Nordic countries, enabled individual-level data linkage between registers and between children and their mothers.

The study is reported as per the Reporting of studies Conducted using Observational Routinely-collected health Data (RECORD) guideline (S1 File). The analytical approach used in this study followed a prospectively defined protocol (S2 File). An adjustment in the protocol was made: addition of an exploratory analysis of congenital heart defects in children with a cardiovascular disease. In the sensitivity analysis excluding Norway, we added an analysis without imputing missing data on smoking. We also added an analysis on a matched sample for type 2 diabetes.

#### CoNARTaS

The CoNARTaS cohort consists of all children born after ART or SC between 1984 and 2015 in Norway, 1985 and 2015 in Sweden, 1990 and 2014 in Finland, and 1994 and 2014 in Denmark. The data were obtained from the national ART registers and medical birth registers (MBRs), all with high quality [24]. Some of the following registers, and the descriptions of them, have been used in an earlier publication about children born after ART [25].

#### MBRs

The MBRs have covered nearly all births in the Nordic countries over several decades. Reporting to the registers is mandatory [26]. In this study, the MBRs provided data on maternal age at delivery, parity, smoking at first antenatal visit, and body mass index (BMI) before pregnancy or in first trimester; child’s year and month of birth, sex, gestational age, and birth weight; and whether the birth was a stillbirth or livebirth. Stillbirth was defined according to national criteria in each country [24].

#### ART registration

In Norway public and private ART clinics report detailed information to the MBR on all ART cycles that result in ongoing pregnancies verified by ultrasound in gestational week 6−7 [24]. In Sweden, all ART treatments leading to deliveries were reported to the Swedish National Board of Health and Welfare with maternal PIN code between 1982 and 2006. Since 2007, all ART clinics in Sweden, public as well as private, report all ART cycles to the Swedish National Quality Register for Assisted Reproduction [27], which has almost 100% completeness. In Finland, no national ART register exists, but ART conception has been recorded at an individual level at delivery as a dichotomous variable in the MBR from 1990 [24]. In Denmark, the national ART register was established in 1994, with mandatory registration of all ART cycles for both public and private ART clinics. Thus, the register has almost 100% coverage [28]. In this study, the national ART registers provided information about treatment (fresh embryo transfer or frozen embryo transfer [FET]), fertilization method (in vitro fertilization [IVF] or intracytoplasmic sperm injection [ICSI]), date of embryo transfer, culture length, date of birth, and own or donated gametes.

#### NPRs

The NPRs include diagnoses for all inpatient care since 2008 in Norway, 1987 in Sweden, 1967 in Finland, and 1977 in Denmark. Outpatient visits in public hospitals and specialized healthcare in private clinics have been included since 2008 in Norway, 2001 in Sweden, and 1998 in Finland. In Denmark, outpatient visits in public hospitals have been registered since 1995, and information about specialized healthcare in private clinics since 2003 [24]. The registers have high coverage rates and high validity, with positive predictive values in the range of 81%–94% for Denmark [29], 75%−99% for common diagnoses in Finland [30], and 80%−95% in Norway [31–34]. In Sweden, the NPR is divided into an inpatient register and an outpatient register. The inpatient register has high coverage rate (99% of all hospital discharges from somatic [including surgery] and psychiatric care are registered in the inpatient register) and a positive predictive value of 85%−95% for most diagnoses [35]. The outpatient register has a considerably lower coverage rate, and for the registered visits about 80% have a principal diagnosis [36]. Primary care treatment is not recorded in the NPRs.

#### NDR

The NDR started in 1996. All individuals in Sweden with diabetes mellitus, except for gestational diabetes, from the age of 18 years are included. Today, the register covers 98% of specialized inpatient care and 92% of outpatient visits [37].

#### SWEDIABKIDS

SWEDIABKIDS started in year 2000 and is incorporated as a part of the NDR. The register includes children and adolescents in Sweden with a diagnosis of diabetes mellitus under the age of 18 years [37].

#### SPDR

The SPDR linked with personal identity number started in July 2005. The SPDR contains all filled prescriptions in Sweden for drugs and includes data about name of drug, Anatomic Therapeutic Chemical (ATC) code, date of prescription, date when the product was collected, and prescription amount [38].

#### BORIS

BORIS was established in 2005. The register includes children under 18 years of age who are treated for obesity at pediatric clinics or in hospital care in Sweden. According to the annual report of BORIS, the coverage ratio increased rapidly from 2008, and from 2013 approximately 80% of children treated for obesity are included in the register [39].

#### CDRs

The CDRs include all individuals who have died, either in the country or abroad, and who were registered in the country at the time of death. The register started 1951 in Norway [40], 1952 in Sweden [41], 1936 in Finland [42], and 1973 in Denmark [43].

#### Statistical bureaus

The statistical bureaus are the central authorities for recording statistics on all individuals in the respective countries and contain information such as educational level, country of birth, and emigration. In Norway, information about emigration comes from the National Population Register, administered by the Norwegian Tax Administration.

### Study population

**Fig 1:**
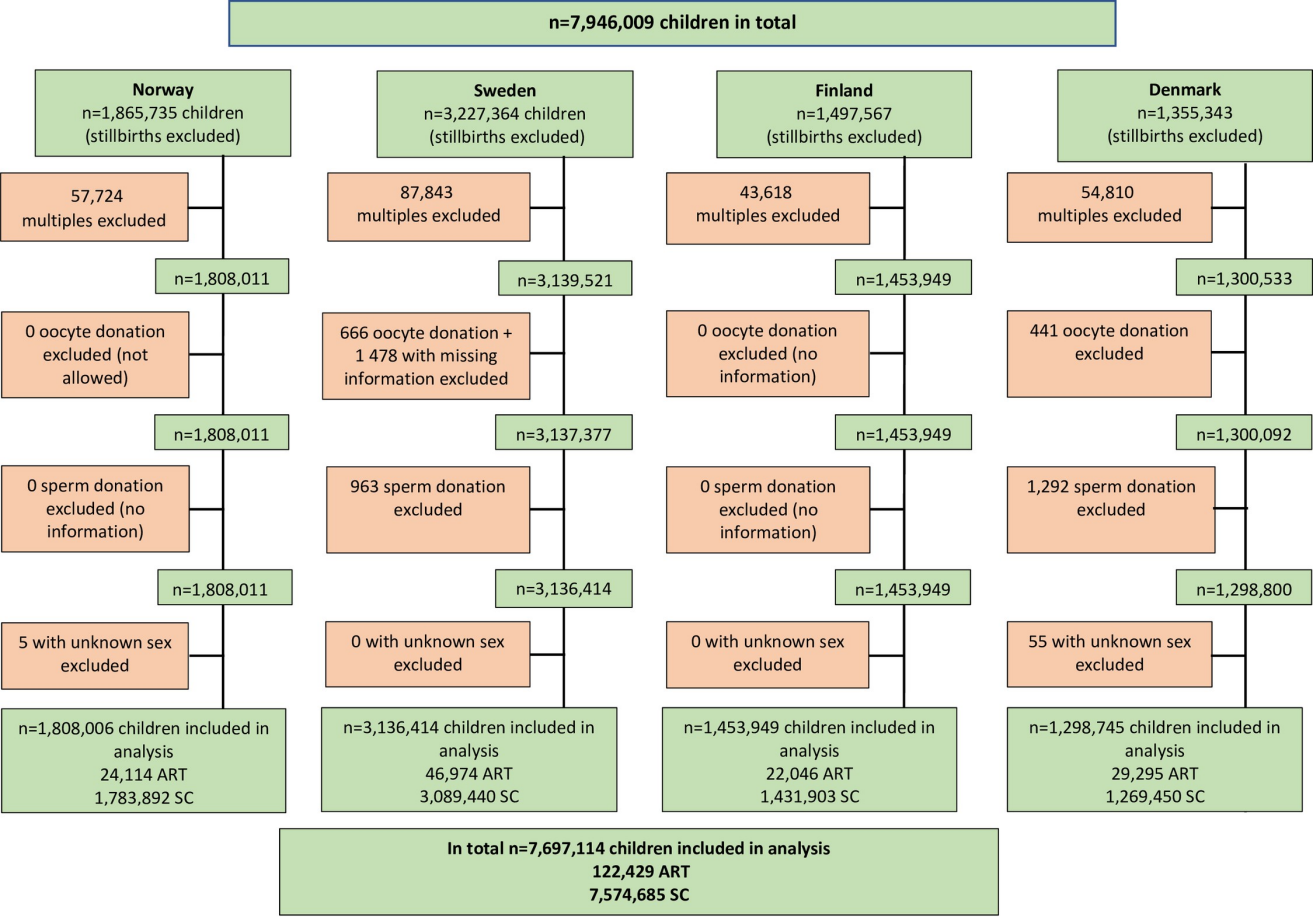
Flowchart of children included in the study. ART, assisted reproductive technology; SC, spontaneous conception.

During the time period from 1 January 1984 to 31 December 2015 in Norway, 1 January 1985 to 31 December 2015 in Sweden, 1 October 1990 to 31 December 2014 in Finland, and 1 January 1994 to 31 December 2014 in Denmark, 7,946,009 children were born (stillbirths excluded). We excluded 243,995 multiple births, 2,585 children born after oocyte donation (OD) or missing information about OD (only from Sweden and Denmark since there was no available information from Finland and OD was not allowed in Norway during the study period), 2,255 children born after sperm donation (only from Sweden and Denmark since there was no available information from Norway and Finland), and 60 children with unknown sex. Consequently, 248,895 children were excluded and 7,697,114 children were included in the study (Fig 1).

### Outcome variables and follow-up

The primary outcomes were any cardiovascular disease, obesity, and type 2 diabetes.

### Definition of cardiovascular disease

“Any cardiovascular disease” was defined if the child had a discharge diagnosis from hospital (inpatient register) with a code for any specific cardiovascular disease in ICD-8, ICD-9, or ICD-10 (S3 File) or a code for any specific cardiovascular disease in the CDRs. Any cardiovascular disease included ischemic heart disease, cardiomyopathy, heart failure, and cerebrovascular disease (hemorrhagic stroke, ischemic stroke, or any other cerebrovascular disease). Ischemic heart disease and cerebrovascular disease were only included if they were principal diagnoses, whereas cardiomyopathy and heart failure could be either principal or secondary diagnoses. Maternal cardiovascular disease was defined according to the same criteria as for the children.

### Definition of obesity

Obesity was defined if the child had a discharge diagnosis from hospital or outpatient visits with a specific code for obesity in ICD-8, ICD-9, or ICD-10 (S3 File). Since obesity is mainly diagnosed at outpatient visits, and outpatient visits were included in the NPRs since 1995 in Denmark, 1998 in Finland, 2001 in Sweden, and 2008 in Norway, we decided to exclude children who were born before these years in each respective country, for the outcome variable obesity. Although children with diabetes born before these dates might have been registered in the outpatient register later in life, the age at onset would be unknown, making it impossible to calculate any hazard ratio (HR). Any child registered in BORIS was also defined as having obesity. Maternal obesity was defined according to the same criteria as for the children, except for appearance in BORIS.

### Definition of type 2 diabetes

Type 2 diabetes in the children was, in Norway, Finland, and Denmark, classified exclusively from data in the NPRs. In these countries, type 2 diabetes was defined if the child had a discharge diagnosis from hospital or outpatient visits with the ICD-10 code for type 2 diabetes (S3 File). Before 1997, the ICD-8 and ICD-9 code for diabetes did not distinguish between type 1 and 2 diabetes. Therefore, children with only an ICD-8 or ICD-9 code for diabetes in the NPRs and no further information on type 1 or 2 diabetes with an ICD-10 code were considered as having type 1 diabetes, since this is the predominant form of diabetes in childhood, and thus not included as events. In Sweden, type 2 diabetes was defined if the child met at least 1 of 4 criteria: (1) discharge diagnosis from hospital or outpatient visits (NPR) with the ICD-10 code for type 2 diabetes (S3 File) (ICD-8 and ICD-9 do not separate type 1 and 2 diabetes); (2) type 2 diabetes diagnosis in SWEDIABKIDS or NDR; (3) diabetes diagnosis of unknown type in SWEDIABKIDS (there is no such code in NDR), treated with no medication or treated with oral antidiabetics (with or without insulin), based on data from SWEDIABKIDS; or (4) diabetes diagnosis of unknown type in SWEDIABKIDS (no such code exists in NDR, see above), treated with no medication or treated, based on data from the SPDR, with oral antidiabetics (≥2 prescriptions of oral antidiabetics [ATC A10B] for males and ≥3 prescriptions for females [for exclusion of gestational diabetes] during the whole study period) with or without insulin (ATC A10A).

All children who were diagnosed with both type 1 and type 2 diabetes were considered as having type 1 diabetes and excluded as events, due to their young age and therefore much greater probability of having type 1 diabetes. Maternal diabetes was defined as any diabetes (type 1 or 2 diabetes). In Norway, Finland, and Denmark this was defined if the mother had a discharge diagnosis from hospital or outpatient visits with the ICD-10 code for type 1 or 2 diabetes (S3 File). In Sweden, any diabetes was defined if the mother was recorded as having type 1 or 2 diabetes in NDR with a date of diagnosis. Date of onset was defined as the first date when the diagnosis was recorded. The end of follow-up was 31 December 2014 in Finland and Denmark and 31 December 2015 in Norway and Sweden.

### Covariates

A fixed set of covariates was selected a priori. Child characteristics were sex, calendar year of birth (continuous variable), and country of birth. Maternal characteristics included age at birth of child (continuous variable), smoking at first antenatal visit (yes or no), highest educational level (basic [≤9 years], secondary [10–12 years], or tertiary [≥13 years]), any cardiovascular disease, obesity, and any diabetes before or at birth of child.

### Ethical approval

Approval for data retrieval was obtained in each country. In Norway, ethical approval was given by the Regional Committee for Medical and Health Research Ethics (REK-Nord, 2010/1909). In Sweden, the Regional Ethical Committee at the University of Gothenburg approved the study (Dnr 214–12, T422–12, T516–15, T233–16, T300–17, T1144–17, T121–18, T1071–18). In Denmark and Finland, ethical approval is not required for register-based studies.

### Statistical analysis

Descriptive statistics are presented as frequency (*n*) and percent for categorical variables and as mean and standard deviation (SD) and/or median and range for continuous variables. Cox proportional hazards models were used to calculate the risk of any cardiovascular disease, obesity, and type 2 diabetes. We used age as the time scale and included each child’s time at risk computed from the date of birth until whichever event occurred first: diagnosis of any cardiovascular disease, obesity, or type 2 diabetes; emigration (not available for Finland); death; or end of the follow-up period (31 December 2014 for Finland and Denmark; 31 December 2015 for Norway and Sweden).

Cardiovascular disease, obesity, and type 2 diabetes were analyzed separately, i.e., if a child was diagnosed with obesity, the child continued to be followed up in the study for the outcomes type 2 diabetes and cardiovascular disease. The same child could occur in several subgroups of cardiovascular disease but only 1 time in “any cardiovascular disease.” We estimated unadjusted and adjusted HRs (aHRs) with 95% confidence intervals (CIs) using Cox regression (maximum likelihood). Corresponding *p-*values are reported, and significance level was set to 5%. In the main analysis, including all countries, adjustment was made for the following variables based on medical knowledge: child sex, child calendar year of birth, child country of birth, maternal age, any maternal cardiovascular disease before or at birth (for the outcome any cardiovascular disease), any maternal obesity before or at birth (for the outcome obesity), and any maternal diabetes before or at birth (for the outcome type 2 diabetes). For the outcome type 2 diabetes, no adjustment for maternal age or child’s country of birth was made, due to there being few events.

Three sensitivity analyses were performed. Since the NPR in Norway first started in 2008 and since there was no information about educational level from Norway and a substantial proportion with missing data on smoking in Norway, we performed a sensitivity analysis in which data from Norway were excluded. In this analysis, adjustments were made for the same variables as in the main analysis and also for maternal highest educational level and maternal smoking at first antenatal visit. Missing data for smoking were imputed as no smoking in the regression analyses. To evaluate what impact this approach had on the results, we also performed analyses in which missing data for smoking were not imputed. In another sensitivity analysis, children born between 1996 and 2005 were analyzed for the outcome any cardiovascular disease (too few events for type 2 diabetes). This analysis was performed because the follow-up time in the total cohort differed considerably between children born after ART and children born after SC, and a potential underestimation of the true association between ART conception and cardiovascular diseases and type 2 diabetes could exist. In this analysis, the same adjustment was made as in the main analysis for any cardiovascular disease. In a third sensitivity analysis, for type 2 diabetes we further investigated the opposite directions of associations from the overall incidence rates and from the Cox regression. To account for the differences in mean follow-up time after ART and SC, we drew a random sample of 12 children born after SC for every child born after ART, matched on year of birth and country.

Finally, 2 subgroup analyses, including data from Norway, Sweden, and Denmark, were performed in which children born after FET were compared to children born after fresh embryo transfer, and children born after ICSI were compared to children born after IVF. In these analyses, adjustment was made for child sex, calendar year of birth, maternal age, maternal any cardiovascular disease (for the outcome any cardiovascular disease), and maternal any obesity before or at birth (for the outcome obesity). The same subgroup analyses were also performed but with data only from Sweden and Denmark. In these analyses, adjustments were made for the same variables as in the subgroup analyses with data from Norway, Sweden, and Denmark and also for maternal highest educational level and maternal smoking at first antenatal visit. We did not adjust for gestational age, birth weight, or birth defects in any of the analyses since these are potential causal pathway characteristics. The analyses were performed in Stata version 15.1.

## Results

In total 7,697,114 children were included in the analysis. Of these, 122,429 children were born after ART and 7,574,685 were born after SC (Fig 1; Table 1). In total, 135 (0.11%) children born after ART and 10,702 (0.14%) children born after SC developed cardiovascular disease (ischemic heart disease, cardiomyopathy, heart failure, or cerebrovascular disease) during the study period. The corresponding values for obesity and type 2 diabetes were 645 (0.65%) and 18 (0.01%), respectively, for children born after ART and 30,308 (0.74%) and 2,919 (0.04%), respectively, for children born after SC (Table 2). A total of 237,379 (2,143 ART and 235,236 SC) children were censored before end of follow-up due to emigration, and 47,442 (637 ART and 46,805 SC) children due to death from other causes.

**Table 1:** Background characteristics of singletons born after ART and spontaneous conception between 1984 to 2015^a^ in Sweden, Norway, Finland, and Denmark, and their mothers.

Characteristic	All countries *n =* 7,697,114		Norway *n =* 1,808,006		Sweden *n =* 3,136,414		Finland *n =* 1,453,949		Denmark *n =* 1,298,745	
	ART *n =* 122,429	SC *n =* 7,574,685	ART *n =* 24,114	SC *n =* 1,783,892	ART *n =* 46,974	SC *n =* 3,089,440	ART *n =* 22,046	SC *n =* 1,431,903	ART *n =* 29,295	SC *n =* 1,269,450
**Child characteristics**										
Calendar year of birth^a^, *n* (%)										
1984–1990	968 (0.8)	1,073,580 (14.2)	477 (2.0)	375,572 (21.1)	461 (1.0)	633,618 (20.5)	30 (0.1)	63,869 (4.5)	0 (0.0)	0 (0.0)
1991–1995	6,531 (5.3)	1,289,711 (17.0)	1,343 (5.6)	290,460 (16.3)	2,818 (6.0)	551,451 (17.9)	1,706 (7.7)	313,763 (21.9)	664 (2.3)	134,001 (10.6)
1996–2000	17,398 (14.2)	1,299,565 (17.2)	2,496 (10.4)	284,106 (15.9)	5,602 (11.9)	420,635 (13.6)	4,407 (20.0)	276,492 (19.3)	4,893 (16.7)	317,969 (25.1)
2001–2005	24,306 (19.9)	1,296,763 (17.1)	3,992 (16.6)	269,009 (15.1)	8,617 (18.3)	454,500 (14.7)	4,574 (20.8)	269,749 (18.8)	7,123 (24.3)	303,349 (23.9)
2006–2010	34,405 (28.1)	1,375,332 (18.2)	7,274 (30.2)	284,711 (16.0)	12,615 (26.9)	505,882 (16.4)	5,622 (25.5)	284,489 (19.9)	8,894 (30.4)	300,158 (23.6)
2011–2015	38,821 (31.7)	1,241,480 (16.4)	8,532 (35.4)	280,434 (15.7)	16,861 (35.9)	523,354 (16.9)	5,707 (25.9)	223,541 (15.6)	7,721 (26.4)	213,973 (16.9)
Birth weight, *n* (%)										
>4,000 g	18,910 (15.5)	1,400,195 (18.5)	3,779 (15.7)	342,675 (19.2)	7,565 (16.1)	576,246 (18.6)	3,413 (15.5)	263,515 (18.4)	4,153 (14.2)	217,759 (17.2)
Low birth weight, <2,500 g	7,008 (5.7)	248,605 (3.3)	1,429 (5.9)	61,381 (3.4)	2,525 (5.4)	98,611 (3.2)	1,187 (5.4)	41,931 (2.9)	1,867 (6.4)	46,682 (3.7)
Very low birth weight, <1,500 g	1,592 (1.3)	45,304 (0.6)	347 (1.4)	11,266 (0.6)	541 (1.2)	16,667 (0.5)	241 (1.1)	7,122 (0.5)	463 (1.6)	10,249 (0.8)
Birth weight (grams), mean (SD)	3,442 (624)	3,541 (565)	3,441 (633)	3,548 (571)	3,457 (612)	3,543 (559)	3,455 (598)	3,552 (542)	3,405 (652)	3,515 (592)
Missing, *n* (%)	298 (0.2)	31,922 (0.4)	13 (0.1)	1,435 (0.1)	123 (0.3)	6,500 (0.2)	8 (0.0)	3,285 (0.2)	154 (0.5)	20,702 (1.6)
LGA, *n* (%)	5,305 (4.3)	355,018 (4.7)	1,035 (4.3)	83,328 (4.7)	2,080 (4.3)	142,628 (4.6)	972 (4.4)	67,953 (4.8)	1,218 (4.2)	61,109 (4.8)
SGA, *n* (%)	5,842 (4.8)	272,604 (3.6)	1,234 (5.1)	72,908 (4.1)	2,222 (4.6)	104,255 (3.4)	876 (4.0)	45,451 (3.2)	1,510 (5.2)	50,119 (4.0)
Gestational age, *n* (%)										
Post-term birth (≥42 weeks)	6,877 (5.6)	536,891 (7.1)	1,458 (6.1)	166,671 (9.3)	3,098 (6.6)	219,554 (7.1)	924 (4.2)	67,522 (4.7)	1,397 (4.8)	83,144 (6.6)
Preterm birth (<37 weeks)	9,630 (7.9)	362,575 (4.8)	1,977 (8.2)	88,156 (4.9)	3,547 (7.6)	151,560 (4.9)	1,738 (7.9)	61,643 (4.3)	2,368 (8.1)	61,216 (4.8)
Very preterm birth (<32 weeks)	1,773 (1.5)	51,101 (0.7)	408 (1.7)	13,750 (0.8)	635 (1.4)	20,169 (0.7)	263 (1.2)	8,164 (0.6)	467 (1.6)	9,018 (0.7)
Extremely preterm birth (<28 weeks)	643 (0.5)	16,716 (0.2)	128 (0.5)	4,719 (0.3)	219 (0.5)	6,393 (0.2)	101 (0.5)	2,761 (0.2)	195 (0.7)	2,843 (0.2)
Gestational age (days), mean (SD)	276 (43.1)	278 (12.9)	277 (92.2)	279 (13.9)	277 (15.1)	278 (12.7)	276 (14.6)	278 (11.9)	276 (16.0)	278 (12.9)
Missing, *n* (%)	358 (0.3)	116,159 (1.5)	109 (0.5)	78,693 (4.4)	25 (0.1)	3,140 (0.1)	46 (0.2)	6,477 (0.5)	178 (0.6)	27,849 (2.2)
Any major birth defect, *n* (%)^b^	5,232 (4.3)	229,754 (3.0)	1,174 (4.9)	58,314 (3.3)	1,699 (3.6)	80,488 (2.6)	958 (4.3)	46,267 (3.2)	1,401 (4.8)	44,685 (3.5)
Congenital heart defect, *n* (%)^c^	1,565 (1.3)	65,806 (0.9)	307 (1.3)	12,535 (0.7)	436 (0.9)	24,569 (0.8)	447 (2.0)	17,913 (1.3)	375 (1.3)	10,799 (0.9)
Cesarean delivery, *n* (%)	32,041 (26.2)	1,095,906 (14.5)	5,719 (23.7)	239,538 (13.4)	12,180 (25.9)	422,116 (13.7)	6,117 (27.8)	218,410 (15.3)	8,025 (27.4)	215,842 (17.0)
Follow-up time (years), mean (SD), median (range)	8.6 (6.2), 7.5 (0.0–31.5)	14.0 (8.6), 13.6 (0.0–32.0)	8.5 (6.5), 7.0 (0.0–31.5)	15.6 (9.2), 15.7 (0.0–32.0)	8.5 (6.4), 7.1 (0.0–30.6)	15.1 (9.2), 15.0 (0.0–31.0)	9.4 (6.3), 8.6 (0.0–24.2)	12.7 (7.4), 12.8 (0.0–25.0)	8.2 (5.4), 7.5 (0.0–20.5)	10.5 (6.1), 10.5 (0.0–21.0)
**Maternal characteristics**										
Age at delivery, (years), mean (SD)	33.9 (4.3)	29.7 (5.2)	33.6 (4.2)	29.2 (5.2)	34.2 (4.2)	29.7 (5.2)	34.0 (4.7)	29.8 (5.3)	33.7 (4.2)	30.1 (4.9)
Primiparous, *n* (%)	82,934 (67.7)	3,163,018 (41.8)	15,329 (63.6)	738,263 (41.4)	33,367 (71.0)	1,310,160 (42.4)	14,711 (66.7)	577,369 (40.3)	19,527 (66.7)	537,226 (42.3)
Smoking at first visit to antenatal clinic, *n* (%)	6,868 (5.6)	964,044 (12.7)	1,204 (5.0)	125,510 (7.0)	1,862 (4.0)	430,120 (13.9)	1,369 (6.2)	219,489 (15.3)	2,433 (8.3)	188,925 (14.9)
Missing, *n* (%)	10,383 (17.8)	1,314,320 (25.1)	5,664 (23.5)	979,902 (55)	2,537 (5.4)	169,567 (5.5)	312 (1.4)	36,405 (2.5)	1,870 (6.4)	128,447 (10.1)
BMI (kg/m^2^) at first visit to antenatal clinic, mean (SD)	24.2 (4.1)	24.1 (4.5)	24.3 (4.3)	24.3 (4.8)	24.4 (3.9)	24.0 (4.3)	24.1 (4.3)	24.3 (4.8)	23.9 (4.3)	24.3 (5.0)
Missing, *n* (%)	41,222 (33.7)	3,716,763 (49.1)	16,299 (67.6)	1,534,660 (86.0)	5,057 (10.8)	662,981 (21.4)	9,545 (43.2)	846,434 (59.1)	10,321 (35.2)	672,688 (53.0)
Highest educational level^d^, *n* (%)										
Basic, ≤9 years	7,363 (6.0)	843,020 (11.1)	NA	NA	3,652 (7.8)	491,066 (15.9)	1,160 (5.3)	155,906 (10.9)	2,551 (8.7)	196,048 (15.4)
Secondary, 10–12 years	33,131 (27.1)	2,316,622 (30.6)	NA	NA	16,044 (34.2)	1,230,765 (39.8)	6,293 (28.5)	590,185 (41.2)	10,794 (36.9)	495,672 (39.1)
Tertiary, ≥13 years	55,333 (45.2)	2,429,890 (32.1)	NA	NA	25,181 (53.6)	1,201,943 (38.9)	14,593 (66.2)	685,812 (47.9)	15,559 (53.1)	542,135 (42.7)
Missing	26,602 (21.7)	1,985,153 (26.2)	24,124 (100)	1,785,535 (100)	2,097 (4.5)	165,666 (5.4)	0.0 (0.0)	0.0 (0.0)	391 (1.3)	35,595 (2.8)
IVF, *n* (%)^e^	56,216 (58.1)		13,672 (61.0)		27,285 (58.1)		NA		15,259 (55.7)	
ICSI, *n* (%)^e^	40,594 (41.9)		8,745 (39.0)		19,689 (41.9)		NA		12,160 (44.4)	
Fresh embryo transfer, *n* (%)^e^	79,816 (81.7)		17,895 (83.6)		36,111 (76.9)		NA		25,810 (88.1)	
Frozen embryo transfer, *n* (%)^e^	17,870 (18.3)		3,522 (16.4)		10,863 (23.1)		NA		3,485 (11.9)	
Number of transferred embryos, mean (SD)^e^										
SET	47,664 (38.9)		10,057 (41.7)		28,289 (60.2)		NA		9,314 (31.8)	
DET	35,539 (29.0)		10,724 (44.5)		7,576 (16.1)		NA		17,237 (58.8)	
TET	2,179 (1.8)		699 (2.9)		7 (0.01)		NA		1,473 (5.0)	
Missing, *n* (%)	37,047 (30.3)		2,634 (10.9)		11,102 (23.6)		22,046 (100)		1,271 (4.3)	
Any CVD before or at birth of child, *n* (%)	217 (0.2)	7,688 (0.1)	26 (0.1)	557 (0.03)	91 (0.2)	3,552 (0.1)	50 (0.2)	1,935 (0.1)	50 (0.2)	1,644 (0.1)
Obesity before or at birth of child, *n* (%)^f^	4,448 (3.6)	168,486 (2.2)	224 (0.9)	7,854 (0.4)	1,321 (2.8)	55,620 (1.8)	206 (0.9)	10,598 (0.7)	2,697 (9.2)	94,414 (7.4)
Any diabetes before or at birth of child, *n* (%)	871 (0.7)	34,828 (0.5)	157 (0.7)	4,120 (0.2)	396 (0.8)	17,925 (0.6)	128 (0.6)	6,123 (0.4)	190 (0.7)	6,660 (0.5)

ART, assisted reproductive technology; BMI, body mass index; CVD, cardiovascular disease; DET, double embryo transfer; ICSI, intracytoplasmic sperm injection; IVF, in vitro fertilization; LGA, large for gestational age; NA, not available; SC, spontaneous conception; SD, standard deviation; SET, single embryo transfer; SGA, small for gestational age; TET, triple embryo transfer.

^a^Children born between 1984 and 2015 in Norway, 1985 and 2015 in Sweden, 1990 and 2014 in Finland, and 1994 and 2014 in Denmark.

^b^Major birth defect, registered during the first year of birth, was defined according to the EUROCAT classification system.

^c^Congenital heart defect was defined if the child had a diagnosis according to ICD-10 codes Q20–Q26 or ICD-9 codes 745–747 (excluding minor defects according to EUROCAT).

^d^Highest educational level ever registered. Information about educational level is missing for Norway.

^e^The variable “number of embryos transferred” has not been in the registers during the whole study period and is completely missing in Finland since information about ART technique is not available in Finland.

^f^The outpatient register started earlier in Denmark, which could be a possible explanation for the much higher prevalence of maternal obesity at birth.

**Table 2:** Frequencies of cardiovascular disease, obesity, and type 2 diabetes in singletons born in Norway, Sweden, Denmark, and Finland between 1984 and 2015^a^.

Outcome	Number (%) (all countries, *n =* 7,700,402)	
	ART, *n =* 122,429	SC, *n =* 7,574,685
**Cardiovascular disease**		
Any cardiovascular disease^b^	135 (0.11)	10,702 (0.14)
Ischemic heart disease	<5^c^	544 (0.01)
Cardiomyopathy	40 (0.03)	2,595 (0.03)
Heart failure	48 (0.04)	3,526 (0.05)
Cerebrovascular disease^d^	55 (0.04)	4,760 (0.06)
Hemorrhagic stroke	12 (0.01)	1,228 (0.02)
Ischemic stroke	31 (0.03)	1,987 (0.03)
**Obesity**	645 (0.65)	30,308 (0.74)
**Type 2 diabetes**	18 (0.01)	2,919 (0.04)

ART, assisted reproductive technology; SC, spontaneous conception.

^a^Children born between 1984 and 2015 in Norway, 1985 and 2015 in Sweden, 1990 and 2014 in Finland, and 1994 and 2014 in Denmark.

^b^Defined as ischemic heart disease and/or cardiomyopathy and/or heart failure and/or cerebrovascular disease. The same child can occur in several subgroups but only 1 time in any cardiovascular disease.

^c^For ethical reasons, data on groups of less than 5 individuals are not presented.

^d^Defined as hemorrhagic stroke and/or ischemic stroke and/or any other cerebrovascular disease.

Table 1 describes sociodemographic and perinatal characteristics of the study population. Mean maternal age at birth was 33.9 (SD 4.3) years for ART mothers and 29.7 (SD 5.2) years for SC mothers. Whereas 67.7% of ART mothers were primiparous, the corresponding value for SC mothers was 41.8%. Smoking was registered in 5.6% of ART mothers and in 12.7% of SC mothers. Preterm birth (<37 gestational weeks) and low birth weight (<2,500 g) occurred in 7.9% and 5.7% of children born after ART and in 4.8% and 3.3% of children born after SC. The number of children born after ART with any major birth defect and a congenital heart defect was 5,232 (4.3%) and 1,565 (1.3%), respectively. The corresponding values for children born after SC were 229,754 (3.0%) and 65,806 (0.9%), respectively. Mean follow-up time was 8.6 (SD 6.2, range 0.0 to 31.5) years for children born after ART, whereas it was 14.0 (SD 8.6, range 0.0 to 32.0) years for children born after SC, reflecting the increased use of ART during the last decades, resulting in shorter follow-up time.

### Main analysis

Table 2 shows the frequencies of cardiovascular disease, obesity, and type 2 diabetes in the study population.

In the unadjusted analysis, children born after ART had a significantly higher risk than children born after SC of any cardiovascular disease, with 12.7 compared to 9.9 per 100,000 person-years at risk (HR 1.24; 95% CI 1.04–1.48; *p =* 0.02). Children born after ART also had a significantly higher risk of being registered with an obesity diagnosis than children born after SC, with 96.8 compared to 93.9 per 100,000 person-years at risk (HR 1.13; 95% CI 1.05–1.23; *p =* 0.002), and type 2 diabetes, with 1.7 compared to 2.7 per 100,000 person-years at risk (HR 1.71; 95% CI 1.08–2.73; *p =* 0.02) (Table 3). After adjustment, there was no significant difference between children born after ART and children born after SC for any cardiovascular disease (aHR 1.02; 95% CI 0.86–1.22; *p =* 0.80) or type 2 diabetes (aHR 1.31; 95% CI 0.82–2.09; *p =* 0.25). For obesity, however, there was still a significant difference between the 2 groups (aHR 1.14; 95% CI 1.06–1.23; *p =* 0.001) (Table 3). HRs for independent covariates, adjusted for in the regression analyses, are illustrated in S1 Fig (cardiovascular disease), S2 Fig (obesity), and S3 Fig (type 2 diabetes).

**Table 3:** Association between ART and cardiovascular disease, obesity, and type 2 diabetes in singletons born in Norway, Sweden, Denmark, and Finland between 1984 and 2015^a^.

Measure	Cardiovascular disease				Obesity				Type 2 diabetes			
	ART, *n =* 122,429	SC, *n =* 7,574,685	ART versus SC HR (95% CI), *p*-value^b^	ART versus SC aHR^c^ (95% CI), *p-*value^b^	ART, *n =* 99,245	SC, *n =* 4,080,483	ART versus SC HR (95% CI), *p-*value^b^	ART versus SC aHR^d^ (95% CI), *p-*value^b^	ART, *n =* 122,429	SC, *n =* 7,574,685	ART versus SC HR (95% CI), *p-*value^b^	ART versus SC aHR^e^ (95% CI), *p-*value^b^
**All countries**												
Number of events (events/100,000 person-years at risk)	135 (12.7)	10,702 (9.9)			645 (96.8)	30,308 (93.9)			18 (1.7)	2,919 (2.7)		
Person-years at risk	1,060,363	108,000,000			666,082	32,300,000			1,060,802	108,000,000		
HR or aHR (95% CI), *p*-value			1.24 (1.04–1.48), *p =* 0.02	1.02 (0.86–1.22), *p =* 0.80			1.13 (1.05–1.23), *p =* 0.002	1.14 (1.06–1.23), *p =* 0.001			1.71^f^ (1.08–2.73), *p =* 0.02	1.31 (0.82–2.09), *p =* 0.25
**All countries except Norway** ^g^												
Number of events (events/100,000 person-years at risk)	104 (12.2)	8,402 (10.5)			629 (102.2)	29,810 (97.8)			15 (1.8)	2,085 (2.6)		
Person-years at risk	855,428	80,200,000			615,485	30,483,788			855,800	80,200,000		
HR or aHR (95% CI), *p*-value			1.07 (0.87–1.31), *p =* 0.51	1.00 (0.82–1.24), *p =* 0.96			1.14 (1.05–1.23), *p =* 0.002	1.21 (1.11–1.31), *p* < 0.001			1.65 (0.99–2.75), *p =* 0.05	1.45 (0.87–2.41), *p =* 0.16

aHR, adjusted hazard ratio; ART, assisted reproductive technology; CI, confidence interval; HR, hazard ratio; SC, spontaneous conception.

^a^Children born between 1984 and 2015 in Norway, 1985 and 2015 in Sweden, 1990 and 2014 in Finland, and 1994 and 2014 in Denmark.

^b^Unadjusted and adjusted HRs with 95% CIs from Cox regression (maximum likelihood), with corresponding *p-*values.

^c^Adjusted for sex, calendar year of birth, child country of birth, maternal age, and maternal any cardiovascular disease at any time before or at birth.

^d^Adjusted for sex, calendar year of birth, child country of birth, maternal age, and maternal obesity at any time before or at birth of child.

^e^Adjusted for sex, calendar year of birth, and maternal any diabetes before or at birth.

^f^The reverse relation between incidence and HR is further explained in a sensitivity analysis.

^g^Without data from Norway because the national patient register in Norway first starts in 2008. Adjusted for the same variables as above for obesity, diabetes, and cardiovascular disease, respectively, and also maternal smoking at first antenatal visit (missing values for smoking were imputed as no smoking) and maternal highest educational level.

### Sensitivity analyses

One sensitivity analysis was performed in which data from Norway were excluded and adjustment for maternal smoking and maternal highest educational level was added. When ART was compared to SC, the results were similar to those of the main analysis (any cardiovascular disease: aHR 1.00; 95% CI 0.82–1.24; *p =* 0.96; obesity: aHR 1.21; 95% CI 1.11–1.31; *p* < 0.001; type 2 diabetes: aHR 1.45; 95% CI 0.87–2.41; *p =* 0.16) (Table 3). In this analysis, missing data for smoking were imputed as no smoking in the regression analysis. To evaluate what impact this approach had on the results, we performed analyses in which missing data for smoking were not imputed. These analyses showed that imputing only changed the results marginally (cardiovascular disease: aHR changed to 0.88; 95% CI 0.70–1.11; *p =* 0.29; obesity: aHR changed to 1.18; 95% CI 1.08 to 1.28 *p* < 0.001; type 2 diabetes: aHR changed to 1.37; 95% CI 0.79 to 2.37; *p =* 0.26). Another sensitivity analysis was performed in which children born between 1996 and 2005 were compared for the outcome any cardiovascular disease. The mean follow-up time in this cohort was 13.7 (SD 3.1, range 0.0 to 20.0) years for children born after ART and 14.4 (SD 3.3, range 0.0 to 20.0) years for children born after SC. The analysis did not find any significant difference between children born after ART and children born after SC, neither in the unadjusted (HR 1.24; 95% CI 0.93–1.66; *p =* 0.15) nor in the adjusted analysis (aHR 1.12; 95% CI 0.84–1.50; *p =* 0.44) (Table 4).

**Table 4:** Analysis of cardiovascular disease^a^ in children born after ART and SC between 1996 and 2005.

Outcome	Number of events (%)		ART versus SC	
	ART, *n =* 41,704	SC, *n =* 2,596,328	HR (95% CI), *p-*value^b^	aHR^c^ (95% CI), *p-*value^b^
Cardiovascular disease	49 (0.12)	2,626 (0.10)	1.24 (0.93 to 1.66), *p =* 0.15	1.12 (0.84 to 1.50), *p =* 0.44

aHR, adjusted hazard ratio; ART, assisted reproductive technology; CI, confidence interval; HR, hazard ratio; SC, spontaneous conception.

^a^Cardiovascular disease defined as ischemic heart disease and/or cardiomyopathy and/or heart failure and/or cerebrovascular disease.

^b^Unadjusted and adjusted HRs with 95% CIs from Cox regression (maximum likelihood), with corresponding *p-*values.

^c^Adjusted for sex, calendar year of birth, child country of birth, maternal age, and maternal any cardiovascular disease at any time before or at birth.

In the sensitivity analysis with a matched sample for type 2 diabetes, the overall incidence rates were consistent with the results from the Cox regression: 1.7/100,000 person-years at risk for children born after ART compared to 1.3/100,000 person-years at risk for children born after SC (unadjusted HR 1.27; 95% CI 0.78–2.07; aHR 1.25; 95% CI 0.77–2.04; *p =* 0.36).

### Subgroup analyses (FET versus fresh embryo transfer, ICSI versus IVF)

In total, 27 (0.15%) children born after FET, 93 (0.12%) children born after fresh embryo transfer, 51 (0.13%) children born after ICSI, and 69 (0.12%) children born after IVF developed cardiovascular disease during the study period. The corresponding values for obesity were 81 (0.50%) for children born after FET, 433 (0.68%) for children born after fresh embryo transfer (Table 5), 219 (0.54%) for children born after ICSI, and 277 (0.49%) for children born after IVF (Table 6). Neither FET nor ICSI was associated with an increased risk of any cardiovascular disease (FET versus fresh embryo transfer: aHR 1.26; 95% CI 0.80–2.00; *p =* 0.32; ICSI versus IVF: aHR 1.12; 95% CI 0.76–1.64; *p =* 0.57) or obesity (FET versus fresh embryo transfer: aHR 1.21; 95% CI 0.95–1.54; *p =* 0.13; ICSI versus IVF: aHR 1.13; 95% CI 0.95–1.36; *p =* 0.17) in the adjusted analyses (Tables 5 and 6). The numbers of children born after FET and ICSI who were diagnosed with type 2 diabetes were too few to be displayed.

**Table 5:** Association between frozen embryo transfer and cardiovascular disease and obesity in singletons born in Norway, Sweden and Denmark^a^ between 1984 and 2015.

Measure	Cardiovascular disease				Obesity			
	FET, *n =* 17,870	Fresh embryo transfer, *n =* 79,823	FET versus fresh HR (95% CI), *p-*value^b^	FET versus fresh aHR^c^ (95% CI), *p-*value^b^	FET, *n =* 17,870	Fresh embryo transfer, *n =* 79,823	FET versus fresh HR (95% CI), *p-*value^b^	FET versus fresh aHR^d^ (95% CI), *p-*value^b^
**All countries** ^**a**^								
Number of events (%)	27 (0.15)	93 (0.12)			81 (0.50)	433 (0.68)		
Person-years at risk	110,371	715,148			82,518	436,568		
HR or aHR (95% CI), *p*-value			1.62 (1.03–2.55), *p =* 0.04	1.26 (0.80–2.00), *p =* 0.32			1.24 (0.98–1.57), *p =* 0.08	1.21 (0.95–1.54), *p =* 0.13
**Analysis of Swedish and Danish data** ^**e**^								
Number of events (%)	21 (0.15)	69 (0.11)			78 (0.54)	422 (0.68)		
Person-years at risk	90,659	557,498			72,754	400,549		
HR or aHR (95% CI), *p*-value			1.62 (0.96–2.74), *p =* 0.07	1.30 (0.75–2.26), *p =* 0.35			1.25 (0.98–1.60), *p =* 0.07	1.22 (0.95–1.58), *p =* 0.12

aHR, adjusted hazard ratio; CI, confidence interval; FET, frozen embryo transfer; HR, hazard ratio.

^a^Without data from Finland since there exists no information about fresh or frozen embryo transfer in Finland.

^b^Unadjusted and adjusted HRs with 95% CIs from Cox regression (maximum likelihood), with corresponding *p-*values.

^c^Adjusted for sex, calendar year of birth, maternal age, and maternal any cardiovascular disease at any time before or at birth.

^d^Adjusted for sex, calendar year of birth, maternal age, and maternal obesity at any time before or at birth.

^e^Without data from Norway since the national patient register in Norway first starts in 2008, and without data from Finland since there exists no information about fresh or frozen embryo transfer in Finland. Adjusted for the same variables as above for obesity and cardiovascular disease, respectively, and also maternal smoking at first antenatal visit (missing values for smoking were imputed as no smoking) and maternal highest educational level.

**Table 6:** Association between ICSI and cardiovascular disease and obesity in singletons born in Norway, Sweden and Denmark^a^ between 1984 and 2015.

Measure	Cardiovascular disease				Obesity			
	ICSI, *n* = 40,594	IVF, *n* = 56,216	ICSI versus IVF HR (95% CI), *p-*value^b^	ICSI versus IVF aHR^c^ (95% CI), *p-*value^b^	ICSI, *n* = 40,594	IVF, *n* = 56,216	ICSI versus IVF HR (95% CI), *p-*value^b^	ICSI versus IVF aHR^d^ (95% CI), *p-*value^b^
**All countries** ^**a**^								
Number of events (%)	51 (0.13)	69 (0.12)			219 (0.54)	277 (0.49)		
Person-years at risk	292,627	527,228			207,966	292,558		
HR or aHR (95% CI), *p*-value			1.36 (0.93–1.99), *p* = 0.12	1.12 (0.76–1.64), *p* = 0.57			1.22 (1.02–1.45), *p* = 0.03	1.13 (0.95–1.36), *p* = 0.17
**Analysis of Swedish and Danish data** ^**e**^								
Number of events (%)	38	51			212 (0.73)	270 (0.74)		
Person-years at risk	234,663	393,349			185,939	267566		
HR or aHR (95% CI), *p*-value			1.27 (0.82–1.99), *p* = 0.29	1.07 (0.68–1.70), *p* = 0.77			1.22 (1.02–1.47), *p* = 0.03	1.12 (0.93–1.36), *p* = 0.23

aHR, adjusted hazard ratio; CI, confidence interval; HR, hazard ratio; ICSI, intracytoplasmic sperm injection; IVF, in vitro fertilization.

^a^Without data from Finland since there exists no information about fresh or frozen embryo transfer in Finland.

^b^Unadjusted and adjusted HRs with 95% CIs from Cox regression (maximum likelihood), with corresponding *p-*values.

^c^Adjusted for sex, calendar year of birth, maternal age, and maternal any cardiovascular disease at any time before or at birth.

^d^Adjusted for sex, calendar year of birth, maternal age, and maternal obesity at any time before or at birth.

^e^Without data from Norway since the national patient register in Norway first starts in 2008, and without data from Finland since there exists no information about ICSI and IVF in Finland. Adjusted for the same variables as above for obesity and cardiovascular disease, respectively, and also maternal smoking at first antenatal visit (missing values for smoking were imputed as no smoking) and maternal highest educational level.

## Discussion

This large, Nordic population-based cohort study included 122,429 singletons born after ART and 7,574,685 singletons born after SC and explored whether there was any difference in the risk of developing cardiovascular disease, obesity, or type 2 diabetes between the 2 groups. The main findings were that children born after ART had no increased risk of cardiovascular disease or type 2 diabetes after adjustment for measured confounders, while for obesity a small but significant increased risk was noticed. For the outcome any cardiovascular disease, the 95% CI was reasonably narrow, excluding effects of a substantial magnitude, while the 95% CI for type 2 diabetes was wide, not excluding clinically meaningful effects.

Overall, the findings from this study give no reason for immediate worries, in contrast to earlier systematic reviews and meta-analyses [19,20], in which significant changes in cardiometabolic risk factors in children born after ART were found. The results from our study are, however, supported by more recent studies of cardiovascular disease in children born after ART. A population-based cohort study from Israel [21], which included 2,603 children born after ART, 1,721 children born after ovulation induction, and 242,187 children born after SC, showed that hospitalizations up to the age of 18 years involving cardiovascular diseases did not differ between the 3 groups. However, there were only 15 hospitalizations for cardiovascular disease in the IVF group, making it difficult to draw any firm conclusions. Another recent study, which included 172 ART and 78 SC individuals, aged 22 to 35 years, compared cardiovascular risk factors such as BMI, blood pressure, total cholesterol, glucose, and carotid intima–media thickness [22]. The results showed no evidence of increased cardiometabolic risk factors in ART-conceived individuals. However, only 36% of the contacted ART individuals agreed to participate, giving a high risk of selection bias. One reason for the diverging results between the studies could be that most studies included in the systematic reviews and meta-analyses [19,20] comprised individuals in early and late childhood, while Juonala et al. [22] examined adults aged 22−35 years. The present study includes children and adults aged 0−32 years, with about 80% of the children born after ART below the age of 15 years. Furthermore, our study, similar to the study of Shiloh et al. [21], explores clinical events of cardiovascular disease and type 2 diabetes while most earlier studies measured markers and risk factors for disease.

The finding that children born after ART had an increased risk of cardiovascular disease in the unadjusted analysis but no increased risk after adjustment for confounders such as maternal age and maternal cardiovascular disease suggests that the ART technique itself does not cause the differences between the groups. Instead, observed differences could be an effect of parental characteristics. However, there is an increased risk of hypertensive disorders in women pregnant after ART, and the risk is higher after FET than after fresh embryo transfer, indicating that the technique also may have an effect [44–48]. There is evidence that children of women with preeclampsia during pregnancy have increased risks of cardiovascular disease during childhood and young adult life. A recently published meta-analysis, including 36 studies of 53,000 individuals, showed that children born to mothers with preeclampsia demonstrate an increase in systolic blood pressure (5.17 mm Hg) and diastolic blood pressure (4.06 mm Hg) and a small increase in BMI (0.36 kg/m^2^), compared to controls [49].

Cardiovascular disease in children has different etiology than cardiovascular disease in adults. Whereas risk factors for ischemic heart disease, heart failure, and stroke in adults are hypertension, smoking, atrial fibrillation, and diabetes, congenital heart disease is an important risk factor for these diseases in children [50–52]. Earlier studies have shown that children born after ART have an increased risk of congenital heart defects [53,54]. In this study, the proportion of children born after ART with a congenital heart defect was 1.3%, whereas the corresponding value for children born after SC was 0.9% (Table 1). We also evaluated the proportion of children with cardiovascular events who were born with a congenital heart defect. It turned out that 28.2% of children born after ART and 17.2% of children born after SC with a cardiovascular disease were born with a congenital heart defect. Thus, congenital heart defects seem to be an important etiology for cardiovascular diseases, especially among children born after ART.

The finding in the present study that there was no significant difference in the risk of developing type 2 diabetes between children born after ART and children born after SC, after adjustment, was based on few events (18 children born after ART with type 2 diabetes) and should therefore be interpreted with caution. In the meta-analysis from 2017 [20], 7 studies examining glucose profiles in 477 children born after ART and 1,852 children born after SC were included. Fasting insulin levels, fasting glucose levels, and Homeostatic Model Assessment for Insulin Resistance (HOMA-IR) levels were evaluated. The results showed that fasting insulin levels in children born after ART were significantly higher, while fasting glucose levels and HOMA-IR levels were comparable between children born after ART and children born after SC. High heterogeneity was however noted throughout the 3 glucose metabolism markers. Another recently published study [55]—of 380 children born after IVF/ICSI and 380 children born after SC, examined at the age of 6–10 years—showed significantly higher fasting blood glucose, serum insulin, and HOMA-IR levels in children born after IVF/ICSI compared to their counterparts born after SC. Thus, the findings from other studies suggest that there could be differences in glucose metabolism between children born after ART and children born after SC. A published register-based study from Sweden, which included 47,938 children born after ART, found an increased risk of type 1 diabetes in children born after ART compared to children born after SC in the unadjusted analysis. After adjustment, however, the association was only significant in children born after FET [25]. In the present study, there were too few events of type 2 diabetes to do any subgroup analysis of FET versus fresh embryo transfer.

The observation of an increased risk of obesity among children born after ART may have several explanations. Since obesity is a diagnosis not causing severe symptoms, the willingness to seek treatment may vary. There is a possibility that parents of children born after ART seek treatment to a greater extent than other parents do, or that children born after ART have other conditions that make them see the doctor more often, such as sequelae from preterm birth. Another complicating factor is the rather late start of the outpatient registers, since obesity mainly is an outpatient diagnosis. Still, several mechanisms may explain why children born after ART could have an increased risk of obesity. Epigenetic and genetic changes associated with ART have been observed in different studies [56–58]. Epigenetic mechanisms have also been associated with obesity [59–61]. Furthermore, it is well known that ART in general is associated with higher rates of preterm birth, low birth weight, and babies being born small for gestational age [62,63], while more recent studies have reported higher risks in children born after FET of being born large for gestational age and macrosomic (≥4,500 g) compared to children born after fresh embryo transfer and SC [46,64,65]. According to the developmental origins of adult health and disease (DOHaD) hypothesis, an abnormal intrauterine environment will make the fetus adapt. Such fetal adaptation may lead to insulin resistance and an increased risk of obesity and metabolic syndrome later in life [66,67]. A substantial increase in FET cycles is noticed worldwide during recent years.

A major strength of this study is the large size and population-based design, making it possible to study diseases that are rare in the ART population, where most individuals are still young. Moreover, the study is based on national registers with almost complete coverage rates and high validity, minimizing missing data and the risk of selection bias. Finally, it was possible to adjust for several relevant confounders, such as maternal cardiovascular disease, obesity, and diabetes.

An important limitation of the study was the relatively short follow-up time, which also was considerably shorter in children born after ART compared to children born after SC. Thus, there might be an underestimation of the true association between ART conception and cardiovascular diseases and type 2 diabetes. Yet, a sensitivity analysis of children born after ART and SC born between 1996 and 2005 did not find any difference in the risk of developing any cardiovascular disease between children born after ART and SC, suggesting no underestimation of the risk of cardiovascular disease in children born after ART. Still, since cardiovascular disease and type 2 diabetes have a much higher incidence and prevalence in older individuals, the results are only generalizable in a young population. Further limitations were differences between countries in the time periods for data collection, ART registration processes, NPR coverage rates, and dataset sources, which may all create variability. An analysis with a 2-level model with country as the higher level might have been appropriate. However, due to the small number of clusters (4 countries), the large number of observations, and the complex frailty models needed to handle multiple levels in survival analysis, we were not able to come up with a model giving stable and reasonable estimates. We only added country as a covariate in the survival analysis, and it should be noted that we might have underestimated the standard errors. Data on outpatient visits were missing in the beginning of the study period, which resulted in the decision to exclude children who were born before the start of the outpatient registers of the NPRs in the respective countries. This exclusion probably resulted in a substantial loss of obesity data. The obesity data also do not capture obese individuals who have not attended inpatient or outpatient clinics, or in whom obesity was not recorded as a diagnosis. Consequently, the definition of obesity cannot be expected to reflect the true rate of obesity in the general population. Furthermore, the large proportion of missing data on maternal BMI made it impossible to adjust for in the analyses. Instead, we used the ICD code for obesity in the adjustments. Paternal characteristics were also missing, and data on ethnicity were not available due to the European General Data Protection Regulation. Lastly, there was also a risk of unmeasured and unknown confounders.

We conclude that children born after ART had no increased risk of cardiovascular disease and type 2 diabetes after adjustment for known confounders, while a small but significant increase of obesity was noticed. For any cardiovascular disease, the 95% CI was reasonably narrow, excluding effects of a substantial magnitude, while the 95% CI for type 2 diabetes was wide, not excluding clinically meaningful effects. The fact that type 2 diabetes and most of the cardiovascular diseases had a limited number of events, and that there was a slightly increased risk of obesity in children born after ART, suggests a need for further studies with longer follow-up. Small differences in a young population could potentially have major consequences in the future.

## Supporting information

S1 Fig Cardiovascular disease: Forest plot of hazard ratios for regression coefficients included in the analysis of cardiovascular disease in children born after ART versus children born after SC. ART, assisted reproductive technology; CVD, cardiovascular disease; SC, spontaneous conception.
(TIFF)

S2 Fig Obesity: Forest plot of hazard ratios for regression coefficients included in the analysis of obesity in children born after ART versus children born after SC. ART, assisted reproductive technology; SC, spontaneous conception.
(TIFF)

S3 Fig Type 2 diabetes: Forest plot of hazard ratios for regression coefficients included in the analysis of type 2 diabetes in children born after ART versus children born after SC. ART, assisted reproductive technology; SC, spontaneous conception.
(TIFF)

S1 File The RECORD statement—checklist of items, extended from the STROBE statement, that should be reported in observational studies using routinely collected health data. (DOCX)

S2 File Prospectively defined research protocol. (DOCX)

S3 File ICD codes used for definition of cardiovascular diseases, obesity, and type 2 diabetes. (DOCX)

## Abbreviations

aHR: adjusted hazard ratio
ART: assisted reproductive technology
BMI: body mass index
CDR: cause of death register
CI: confidence interval
CoNARTaS: Committee of Nordic Assisted Reproductive Technology and Safety
FET: frozen embryo transfer
HR: hazard ratio
ICD: International Statistical Classification of Diseases and Related Health Problems
ICSI: intracytoplasmic sperm injection
IVF: in vitro fertilization; MBR, medical birth register
NDR: Swedish National Diabetes Register
NPR: national patient register
SC: spontaneous conception
SD: standard deviation
SPDR: Swedish Prescribed Drug Register
