# Supplementary figures and images for: Cardiovascular disease, obesity, and type 2 diabetes in children born after assisted reproductive technology: A population-based cohort study
Source: PLoS Med. 2021 Sep 7;18(9):e1003723. doi: 10.1371/journal.pmed.1003723 (PMC8423242; doi:10.1371/journal.pmed.1003723)

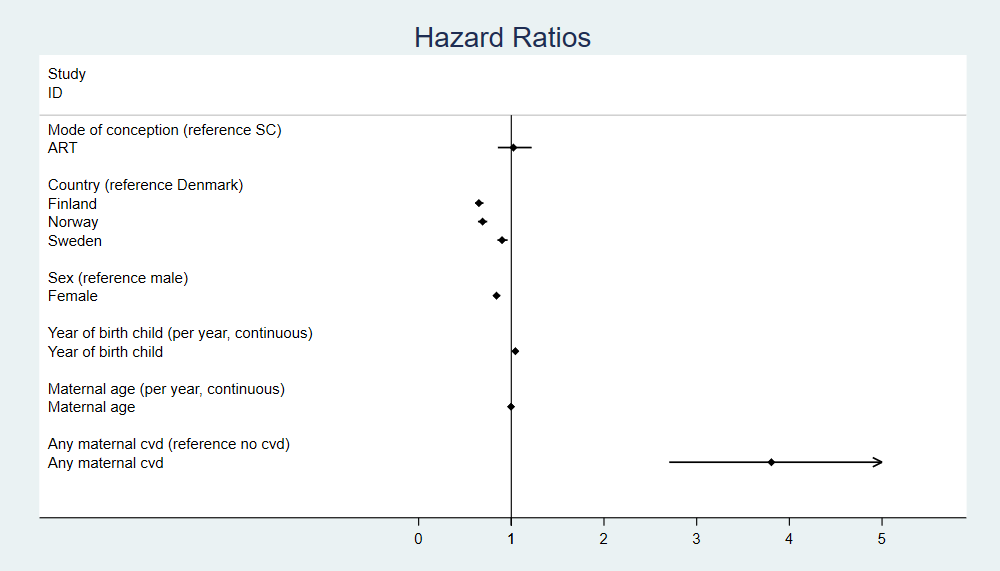

Supplement: S1 Fig — ART, assisted reproductive technology; CVD, cardiovascular disease; SC, spontaneous conception. (TIFF) [file pmed.1003723.s001.tiff]

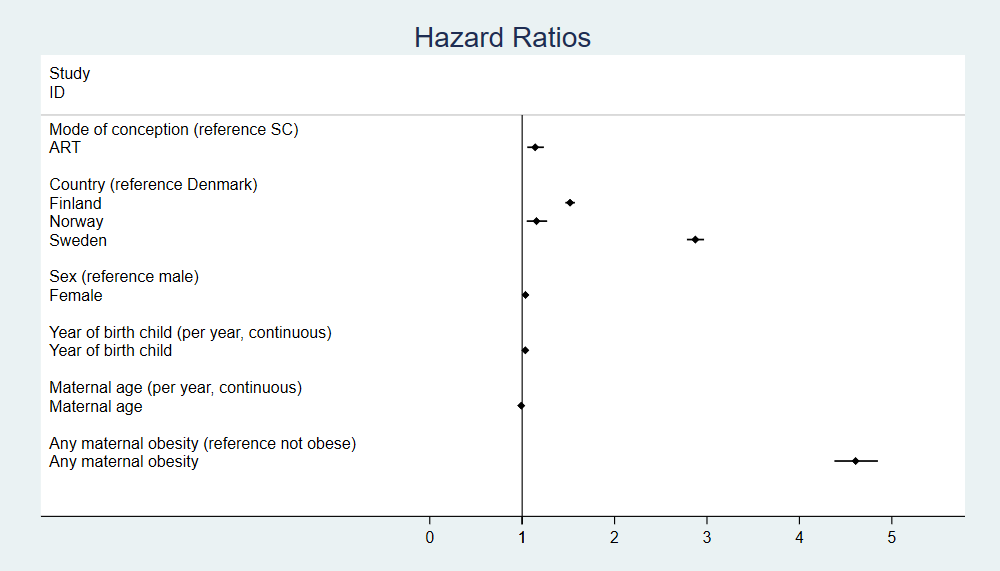

Supplement: S2 Fig — ART, assisted reproductive technology; SC, spontaneous conception. (TIFF) [file pmed.1003723.s002.tiff]

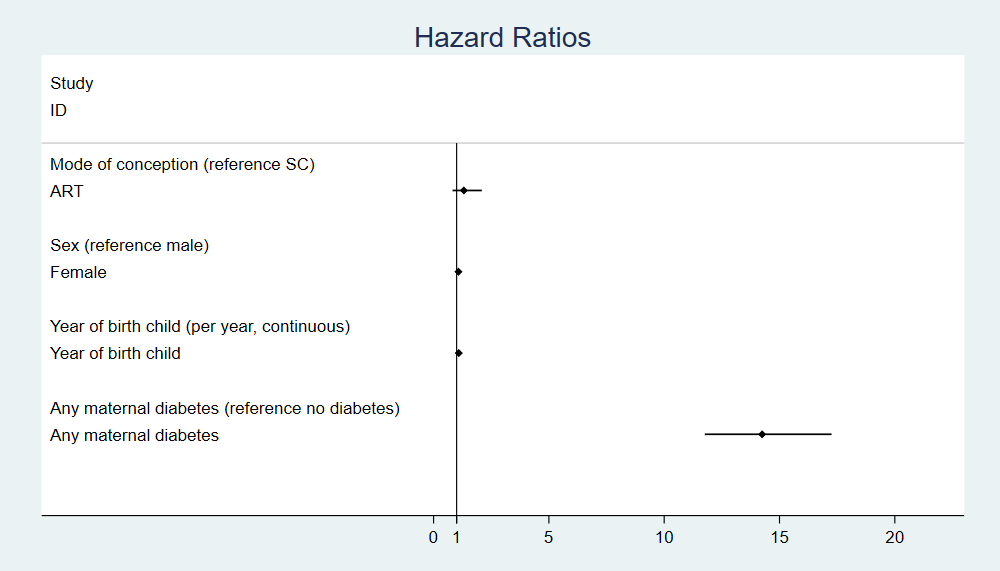

Supplement: S3 Fig — ART, assisted reproductive technology; SC, spontaneous conception. (TIFF) [file pmed.1003723.s003.tiff]
