# Supplementary material for: Cardiovascular disease, obesity, and type 2 diabetes in children born after assisted reproductive technology: A population-based cohort study
Source: PLoS Med. 2021 Sep 7;18(9):e1003723. doi: 10.1371/journal.pmed.1003723 (PMC8423242; doi:10.1371/journal.pmed.1003723)
Supplement: S2 File — (DOCX) [file pmed.1003723.s005.docx]

S2. **Research Protocol/ CoNARTaS/ Cardiovascular, Diabetes type 2 and Obesity project**

Emma Norrman 191207-UBW191211

**Research question**

Do children born after ART have an increased risk of cardiovascular diseases, diabetes type 2 or obesity?

**Background**

Assisted reproductive technology (ART) is associated with higher rates of preterm birth, low birth weight and babies being born small for gestational age,^1^ which are risk factors for future metabolic syndrome.^2^ Furthermore, higher risks of hypertensive disorders in pregnancy are noted after ART, especially after frozen embryo transfer ^3,4^While the maternal and neonatal outcome of ART is well documented, large studies on long-term follow-up are scarce. Some studies raise concerns as to whether fertility treatments may influence long-term morbidity in ART children. Elevated blood pressure and altered glucose metabolism have been found after ART both in animal studies and in a few studies in children.^5-11^ Additionally, there is evidence that offspring of preeclamptic pregnancies have increased risks of cardiovascular disease during childhood and young adult life^12^.The use of ART has increased significantly during the last decades with more than 390,000 children born worldwide annually^13^ and the total number of ART children now exceeding seven million.^14^ By combining high-quality Nordic registries we have the opportunity to study the long-term morbidity of ART children.

**Methods**

We will use the CoNARTaS database which includes several nationwide health data registries as well as quality registries comprising all singletons born after ART and spontaneous conception between 1994 and 2015 in Denmark, 1990 and 2015 in Finland, 1984 and 2015 in Norway and 1985 and 2015 in Sweden.

*Inclusion criteria:* all live-born singletons born after ART and spontaneous conception during the study period.

ART singletons will be identified from the national ART registries and the Medical Birth Registries (MBR). Singletons born after spontaneous conception will be identified from the MBR.

*Exclusion criteria:* Children from multiple pregnancies, stillbirths, children born after oocyte donation.

**The following national registries will be used:**

Sweden: MBR, National Patient Registry In patient (IP) and outpatient (OP), Cause of Death Registry, data from Statistics Sweden (SCB), BORIS and Prescribed Drug Registry, SWEDIABKIDS, NDR

Norway; MBR, National Patient Registry from 2008, Cause of Death Registry

Denmark**:** MBR, National Patient Registry, NDR. Cause of Death Registry

Finland**:** From MBR, National Patient Registry,

**Variables included:**

**For the offspring:**

**Offspring demographics**

**Basic file**: Plurality, sex, birthweight, gestational age (SGA/LGA to be calculated), Apgar score, malformation, neonatal diagnoses (?), date and cause of death. Date of emigration/immigration.

**Cause of death registry (from basic file)** Date and cause of death

**Offspring outcomes**

**From the national patient registry (NPR) (IP and OP) (principal diagnosis or any diagnosis + date), CDR (date), BORIS, SWEDIABKIDS, NDR:**

|  | **Sources** | **Code position** | **ICD 10** | **ICD 9** | **ICD8** |
| --- | --- | --- | --- | --- | --- |
| **Acute coronary events = non-fatal MI + fatal CHD events**  **I21=acute myocardial infarction** | NPR-IP + CDR | Principal diagnosis (NPR-IP) or underlying cause of death (DR) | NPR: i21  DR: i20-i25 | NPR: 410  DR: 410-414 | NPR: 410  DR: 410-414 |
| **Acute MI (AMI)= = non-fatal MI + fatal AMI** | NPR-IP + DR | Principal diagnosis (NPR-IP) or underlying cause of death (DR) | NPR: i21  DR: I21 | NPR: 410  DR: 410 | NPR: 410  DR: 410 |
| **Ischemic heart disease (IHD)** | NPR-IP + DR | Principal diagnosis (NPR-IP) or underlying cause of death (DR). | NPR: i20-I25  DR: i20-I25 | NPR: 410-14  DR: 410-414 | NPR: 410-414  DR: 410-414 |
|  |  |  |  |  |  |
| **Cardiomyopathy** | NPR-IP +DR | Any position (NPR-IP or DR) | i42, i43 | 425 | 425 |
| **Heart failure** | NPR-IP + DR | Any position (NPR-IP or DR) | i50  2551 code | 428  1263 code | 427,00; 427,10  158+1 code |
| **Cerebrovascular disease (CVL)** | NPR-IP + DR | Principal diagnosis (NPR-IP) or underlying cause of death (DR). | i60-I69 | 430 – 438 | 430,00-438,99 |
| **Hemorrhagic stroke** | NPR-IP + DR | Principal diagnosis (NPR-IP) or underlying cause of death (DR). | i61 | 431 | 431 |
| **Ischemic stroke** | NPR-IP + DR | Principal diagnosis (NPR-IP) or underlying cause of death (DR). | i63; i64 | 434, 436 | 433, 434, 436 |
| **Obesity** | NPR-IP and NPR-OP | Sweden:  Any position of E66 (NPR-IP or NPR-OP) or exists in BORIS, or with a surgical procedure for obesity JDF | E66 | 278A, 278B | 277,99 |
| **Diabetes type 2** | NPR-IP and NPR-OP | Any position (NPR-IP or NPR-OP)  SWEDIABKIDS,  Or NDR according to specification and hirerarki | E11 | 250  Not used, does not separate type 1 or 2 | 250  Not used, does not separate type 1 or 2 |

**NPR = national patient registry including outpatient registry (outpatient data available after 2001 in Sweden)**

**NPR-IP = inpatient registry only (= in-hospital), NPR-OP= outpatient register**

**DR=death registry**

**Acute coronary events, AMI (acute myocardial infarction), IHD (ischemic heart disease), stroke is identified as:**

1. Only inpatient ward or death (outpatient diagnosis not relevant since not the first contact)

2. Only as main diagnosis or underlying cause of death– if secondary diagnosis it might be a so called type 2 infarct i.e as a complication to some other disease , for example sepsis, arythmia, . Reasonable to handle stroke in a similar way.

3.Heart failure is defined only as inpatient diagnosis, main or secondary diagnosis. Might occur in any ICD positiion. Heart failure in outpatient registry is not validated, neither death in heart failure.

4.Cardiomyopathy: as heart failure, but as cause of death without earlier diagnosis maybe more valid, ( for example sudden death in an earlier heart healthy patient).

5. Cerebrovascular disease (CVL) includes also subarchnoidal hemorrhage (SAH; I60, 430) and some other uncommon diagnoses, we probably should restrict.

It is reasonable to separate ischemic and hemorrhagic stroke.

**From the Pharmaceutical registry: ATC codes + date of expedition**

Cardiovascular: C01-C04, C06-C10

Diabetes: A10A, A10B

**For obesity from BORIS (Sweden)**

**For the mothers:**

**From Basic file**

ART yes/no, IVF/ ICSI, fresh, frozen, origin oocyte = own, singleton

**From basic file:** Age at birth, parity, BMI (length and weight), smoking during pregnancy, pre-gestational or gestational diabetes, hypertensive disorders in pregnancy (HDP), mode of delivery.

Education for Sweden is in a separate file at Statistics Denmark (DST). Country of birth needs to be analyzed in the different countries separately (for example data from SCB in Sweden and DST in Denmark)

**From National Patient Registry (IP and IP) (diagnosis + date) registered at birth of child.**

**Any I diagnosis according to specification in table below, not specific groups of I diagnoses.**

**For diabetes only NDR data for Sweden (1. any registration in NDR or 2. any registration – DM type 1 (from diabetes paper)= DM type 2)**

|  | **Sources** | **Code position** | **ICD 10** | **ICD 9** | **ICD8** |
| --- | --- | --- | --- | --- | --- |
| **Acute coronary events = non-fatal MI + fatal CHD events**  **I21=acute myocardial infarction** | NPR-IP + DR | Principal diagnosis (NPR-IP) or underlying cause of death (DR) | NPR: i21  DR: i20-i25 | NPR: 410  DR: 410-414 | NPR: 410  DR: 410-414 |
| **Acute MI (AMI)= = non-fatal MI + fatal AMI** | NPR-IP + DR | Principal diagnosis (NPR-IP) or underlying cause of death (DR) | NPR: i21  DR: i21 | NPR: 410  DR: 410 | NPR: 410  DR: 410 |
| **Ischemic heart disease (IHD)** | NPR-IP + DR | Principal diagnosis (NPR-IP) or underlying cause of death (DR). | NPR: i20-i25  DR: i20-i25 | NPR: 410-14  DR: 410-414 | NPR: 410-414  DR: 410-414 |
|  |  |  |  |  |  |
| **Cardiomyopathy** | NPR-IP +DR | Any position (NPR-IP or DR) | i42, i43 | 425 | 425 |
| **Heart failure** | NPR-IP + DR | Any position (NPR-IP or DR) | i50  2551 code | 428  1263 code | 427,00; 427,10  158+1 code |
| **Cerebrovascular disease (CVL)** | NPR-IP + DR | Principal diagnosis (NPR-IP) or underlying cause of death (DR): | i60-I69 | 430 – 438 | 430,00-438,99 |
| **Hemorrhagic stroke** | NPR-IP + DR | Principal diagnosis (NPR-IP) or underlying cause of death (DR). | i61 | 431 | 431 |
| **Ischemic stroke** | NPR-IP + DR | Principal diagnosis (NPR-IP) or underlying cause of death (DR). | i63; i64 | 434, 436 | 433, 434, 436 |
| **Obesity** | NPR-IP and NPR-OP | Sweden:  Any position of E66 (NPR-IP or NPR-OP), or with a surgical procedure for obesity | E66 | 278A, 278B | 277,99 |
| **Diabetes type 2** | NPR-IP and NPR-OP  NDR (only NDR for Sweden) | Any position (NPR-IP or NPR-OP)  Or NDR/DDK according to specification and hierarchy | E11 | 250  Not used, does not separate type 1 or 2 | 250  Not used, does not separate type 1 or 2 |

**NPR = national patient registry including outpatient registry (outpatient data available after 2001)**

**NPR-IP = inpatient registry only (= in-hospital), NPR-OP= outpatient register**

**CDR=cause of death registry.**

**Please see instructions above for Acute coronary events, AMI (acute myocardial infarction), IHD (ischemic heart disease), stroke.**

**For the fathers:**

**From basic file:** Age at birth, education, country of birth

Education for Sweden is in a separate file at Statistics Denmark. Country of birth needs to be analyzed in the different countries separately (for example data from SCB in Sweden and DST in Denmark)

.

**From National Patient Registry (IP and IP) (diagnosis + date) registered at birth of child.**

**Any I diagnosis according to specification in table below, not specific groups of I diagnoses.**

**For diabetes only NDR data for Sweden (1. any registration in NDR or 2. any registration in NDR– DM type 1 (from diabetes paper)= DM type 2)**

|  | **Sources** | **Code position** | **ICD 10** | **ICD 9** | **ICD8** |
| --- | --- | --- | --- | --- | --- |
| **Acute coronary events = non-fatal MI + fatal CHD events**  **I21=acute myocardial infarction** | NPR-IP + DR | Principal diagnosis (NPR-IP) or underlying cause of death (DR | NPR: i21  DR: i20-i25 | NPR: 410  DR: 410-414 | NPR: 410  DR: 410-414 |
| **Acute MI (AMI)= = non-fatal MI + fatal AMI** | NPR-IP + DR | Principal diagnosis (NPR-IP) or underlying cause of death (DR) | NPR: i21  DR: i21 | NPR: 410  DR: 410 | NPR: 410  DR: 410 |
| **Ischemic heart disease (IHD)** | NPR-IP + DR | Principal diagnosis (NPR-IP) or underlying cause of death (DR). | NPR: i20-i25  DR: i20-i25 | NPR: 410-14  DR: 410-414 | NPR: 410-414  DR: 410-414 |
|  |  |  |  |  |  |
| **Cardiomyopathy** | NPR-IP +DR | Any position (NPR-IP or DR) | i42, i43 | 425 | 425 |
| **Heart failure** | NPR-IP + DR | Any position (NPR-IP or DR) | i50  2551 code | 428  1263 code | 427,00; 427,10  158+1 code |
| **Cerebrovascular disease (CVL)** | NPR-IP + DR | Principal diagnosis (NPR-IP) or underlying cause of death (DR). | i60-i69 | 430 – 438 | 430,00-438,99 |
| **Hemorrhagic stroke** | NPR-IP + DR | Principal diagnosis (NPR-IP) or underlying cause of death (DR). | i61 | 431 | 431 |
| **Ischemic stroke** | NPR-IP + DR | Principal diagnosis (NPR-IP) or underlying cause of death (DR). | i63; i64 | 434, 436 | 433, 434, 436 |
| **Obesity** | NPR-IP and NPR-OP | Sweden:  Any position of E66 (NPR-IP or NPR-OP) or exists in BORIS, or with a surgical procedure for obesity JDF | E66 | 278A, 278B | 277,99 |
| **Diabetes type 2** | NPR-IP and NPR-OP  NDR (only NDR for Sweden) | Any position (NPR-IP or NPR-OP)  Or NDR/DDK according to specification and hierarchy | E11 | 250  Not used, does not separate type 1 or 2 | 250  Not used, does not separate type 1 or 2 |

**NPR = national patient registry including outpatient registry (outpatient data available after 2001)**

**NPR-IP = inpatient registry only (= in-hospital), NPR-OP= outpatient register**

**DR=death registry**

**Please see instructions above for Acute coronary events, AMI (acute myocardial infarction), IHD (ischemic heart disease), stroke.**

**Definition of type 2 diabetes (with Swedish codes) for children:**

**1**) Type 2 diabetes diagnosis in SWEDIABKIDS (code 2) or NDR (code 2)

**2)** Diabetes diagnosis of unknown type (code 12 in SWEDIABKIDS, there is no such code in NDR), treated with no medication or treated with oral antidiabetics (with or without insulin) in SWEDIABKIDS (code 1, 2, or 4 for treatment) or NDR (but there are no diagnosis of unknown type)

**3)** Diabetes diagnosis of unknown type in SWEDIABKIDS (code 12) or NDR (no such code exists, see above), treated with no medication or treated (data from prescription database) with oral antidiabetics (≥2 prescriptions of oral antidiabetics [ATC A10 B], for males and ≥3 prescriptions for females [for exclusion of gestational diabetes] during the whole study period) with or without insulin (ATC A10A)

**4)** Hospital discharge diagnosis from hospital or outpatient visits (NPR-IP or NPR-OP) with Type 2 diabetes (ICD10: E11, ICD 9 does not separate Type 1 or 2 DM).

**Covariates**

Year of birth, sex, parity (primiparous/multiparous), maternal and paternal country of origin, maternal and paternal educational level and age at birth. Maternal and paternal diabetes, cardiovascular disease in parents, obesity in parents (all parental morbidity should be registered before or at birth of child)

**Statistical Analysis**

Descriptive statistics will be given by mean, standard deviation (SD) and median (range) for continuous variables and by frequency (n) and percent for categorical variables. The risk of cardiovascular disease, obesity and type 2 diabetes will be estimated as hazard ratios (HRs) using Cox proportional hazards models. We will use age as the time scale and include each child´s time at risk computed from the date of birth until whichever event comes first: diagnosis of cardiovascular disease, obesity or type 2 diabetes, emigration, death, or end of the follow-up period. We will choose a fixed set of covariates and adjust for them. We will estimate crude and adjusted HRs and 95% confidence intervals (CIs). Significance level will be set to 5%.

**Ethical Approval**

**Sweden:** Dnr 214-12, T422-12, T516-15, T233-16, T300-17, T1144-17, T121-18, T1071-18,

**Trial registration number:** ISRCTN11780826

**References**

1.Pinborg A, Wennerholm UB, Romundstad LB, et al. Why do singletons conceived after assisted reproduction technology have adverse perinatal outcome? Systematic review and meta-analysis. Hum Reprod Update 2013;19(2):87-104.

2. Barker DJ, Hales CN, Fall CH, Osmond C, Phipps K, Clark PM. Type 2 (non-insulin-dependent) diabetes mellitus, hypertension and hyperlipidemia (syndrome X): relation to reduced fetal growth. Diabetologia 1993;36(1):62-67.

3. Opdahl S, Henningsen AA, Tiitinen A, Bergh C, Pinborg A, Romundstad PR, Wennerholm UB, Gissler M, Skjaerven R, Romundstad LB. Risk of hypertensive disorders in pregnancies following assisted reproductive technology: a cohort study from the CoNARTaS group. Hum Reprod 2015;30:1724-31

4. Ernstad E, Wennerholm U-B, Khatibi A, Petzold M, Bergh C. Neonatal and maternal outcome after frozen embryo transfer: Increased risks in programmed cycles. AJOG 2019; 221: 126.e1-126.e18

5. Ceelen M, van Weissenbruch MM, Prein J, et al. Growth during infancy and early childhood in relation to blood pressure and body fat measures at age 8-18 years of IVF children and spontaneously conceived controls born to subfertile parents. Hum Reprod 2009;24(11):2788-2795.

6. Chen M, Wu L, Zhao J, et al. Altered glucose metabolism in mouse and humans conceived by IVF. Diabetes 2014;63(10):3189-3198.

7. Hart R and Norman RJ. The longer-term health outcomes for children born as a result of

IVF treatment: Part I--General health outcomes. *Hum Reprod Update* 2013a: **19**; 232-243.

8. Guo XY, Liu XM, Jin L, et al. Cardiovascular and metabolic profiles of offspring conceived by assisted reproductive technologies: a systematic review and meta-analysis. Fertil Steril 2017;107(3):622-631.e625.

9. Vrooman LA, Bartolomei MS. Can assisted reproductive technologies cause adult-onset disease? Evidence from human and mouse. Reproductive toxicology (Elmsford, NY) 2017;68:72-84.

10. Meister TA, Rimoldi SF, Soria R, et al. Association of Assisted Reproductive Technologies With Arterial Hypertension During Adolescence. J Am Coll Cardiol. 2018;72(11):1267-1274.

11. Scherrer U, Rexhaj E, Allemann Y, et al. Cardiovascular dysfunction in children conceived by assisted reproductive technologies. Eur Heart J. 2015 Jul 1;36(25):1583-9.

12. Andraweera P and Lassi. Cardiovascular Risk Factors in Offspring of Preeclamptic Pregnancies – Systematic Review and Meta-Analysis. J Pediatr 2019;208:104-13.

13.Adamson GD, de Mouzon J, Chambers GM, et al. International committee for monitoring assisted reproductive technology: world report on assisted reproductive technology, 2011. Fertil Steril 2018;110:1067-1080

14. Adamson G, Dyer S, Chambers G, et al. International Committee for Monitoring Assisted Reproductive Technologies (ICMART) World Report on ART, 2014 and The International Glossary on Infertility and Fertility Care, 2017. ICMART World Report 2014 2017.
