# Supplementary material for: Cardiovascular disease, obesity, and type 2 diabetes in children born after assisted reproductive technology: A population-based cohort study
Source: PLoS Med. 2021 Sep 7;18(9):e1003723. doi: 10.1371/journal.pmed.1003723 (PMC8423242; doi:10.1371/journal.pmed.1003723)
Supplement: S3 File — (DOCX) [file pmed.1003723.s006.docx]

|  | **Sources** | **Code position** | **ICD 10** | **ICD 9** | **ICD8** |
| --- | --- | --- | --- | --- | --- |
| Ischemic heart disease (IHD) | NPR-IP + CDR | Principal diagnosis (NPR-IP) or underlying cause of death (CDR). | NPR: I20-I25  CDR: I20-I25 | NPR: 410-414  CDR: 410-414 | NPR: 410-414  CDR: 410-414 |
| Cardiomyopathy | NPR-IP + CDR | Any position (NPR-IP or underlying cause of death (CDR) | I42, I43 | 425 | 425 |
| Heart failure | NPR-IP + CDR | Any position (NPR-IP or underlying cause of death (CDR) | I50  2551 code | 428  1263 code | 427,00; 427,10  158+1 code |
| Cerebrovascular disease | NPR-IP + CDR | Principal diagnosis (NPR-IP) or underlying cause of death (CDR). | I60-I69 | 430 – 438 | 430,00-438,99 |
| Hemorrhagic stroke | NPR-IP + CDR | Principal diagnosis (NPR-IP) or underlying cause of death (CDR). | I61 | 431 | 431 |
| Ischemic stroke | NPR-IP + CDR | Principal diagnosis (NPR-IP) or underlying cause of death (CDR). | I63; I64 | 434, 436 | 433, 434, 436 |
| Obesity | NPR-IP and NPR-OP | Sweden:  Any position of E66 (NPR-IP or NPR-OP) or exists in BORIS, or with a surgical procedure for obesity JDF | E66 | 278A, 278B | 277,99 |
| Diabetes type 2 | NPR-IP and NPR-OP | Any position (NPR-IP or NPR-OP)  SWEDIABKIDS,  Or NDR according to specification and hierarchy | E11 | 250  Not used for the children, does not separate type 1 or 2 | 250  Not used for the children, does not separate type 1 or 2 |
| Diabetes type 1 (only used for mothers to create “any diabetes”) | NPR-IP and NPR-OP | Any position (NPR-IP or NPR-OP)  SWEDIABKIDS,  Or NDR according to specification and hierarchy | E10 | 250 | 250 |

**S3 File.** ICD-codes used for definition of cardiovascular diseases, obesity and type 2 diabetes.

ICD, International Statistical Classification of Diseases and Related Health Problems; CDR, Cause of Death Register; NPR-IP, National Patient Register - In-Patient Register; NPR-OP, National Patient Register- Out-Patient Register
